# Decreased β-Cell Function Is Associated with Reduced Skeletal Muscle Mass in Japanese Subjects without Diabetes

**DOI:** 10.1371/journal.pone.0162603

**Published:** 2016-09-09

**Authors:** Satoshi Sakai, Keiji Tanimoto, Ayumi Imbe, Yuiko Inaba, Kanako Shishikura, Yoshimi Tanimoto, Takahisa Ushiroyama, Jungo Terasaki, Toshiaki Hanafusa

**Affiliations:** 1 Department of Internal Medicine (I), Osaka Medical College, Takatsuki City, Osaka, 569-8686, Japan; 2 Health Science Clinic, Osaka Medical College, Takatsuki City, Osaka, 569-1121, Japan; Universita degli Studi Magna Graecia di Catanzaro Scuola di Medicina e Chirurgia, ITALY

## Abstract

**Background:**

Decreased insulin secretion has a great impact on the incidence of type 2 diabetes in Japanese subjects. It is not clear whether β-cell function is related to muscle mass in subjects without diabetes. We investigated the relationship between β-cell function and skeletal muscle mass in Japanese subjects without diabetes.

**Methods:**

The study included 1098 subjects (538 men and 560 women) aged 40 to 79 years, without diabetes (fasting glucose lower than 126 mg/dL and glycosylated hemoglobin lower than 6.5%), who consulted Osaka Medical College Health Science Clinic for a medical examination. Appendicular muscle mass was measured by bioelectrical impedance analysis. Appendicular muscle mass index was calculated as appendicular muscle mass divided by height squared (kg/m^2^). The homeostatic model assessment of β-cell function was used to assess β-cell function. The homeostatic model assessment of insulin resistance was used as a measure of insulin resistance. The association between appendicular muscle mass index and clinical parameters of β-cell function and insulin resistance was examined.

**Results:**

Log-transformed homeostatic model assessment of β-cell function and Log-transformed homeostatic model assessment of insulin resistance showed a normal distribution. In both men and women, there was a significant positive correlation between appendicular muscle mass index and clinical parameters of β-cell function and insulin resistance. Tertile analysis, following stratification according to appendicular muscle mass index, found that low appendicular muscle mass index was significantly associated with the Log homeostatic model assessment of β-cell function and Log-transformed homeostatic model assessment of insulin resistance.

**Conclusion:**

This study shows that decreased β cell function is associated with reduced skeletal muscle mass in Japanese subjects without diabetes.

## Introduction

The number of patients with type 2 diabetes is increasing globally [1], and prevention of diabetes is a worldwide concern. Decreased insulin secretion and increased insulin resistance are related to the onset of diabetes. In Japanese subjects, decreased insulin secretion has a greater impact on the incidence of type 2 diabetes than increased insulin resistance [2].

In 1985, Matthews et al. advocated the homeostatic model assessment (HOMA), which estimated β-cell function (HOMA-β) and insulin resistance (HOMA-IR) based on fasting glucose and insulin levels [3]. It has been reported that HOMA-β correlated with β-cell function evaluated by an oral glucose tolerance test or hyperglycemic clamp test [4,5]. Low HOMA-β was associated with impaired glucose tolerance or diabetes in 1449 Mexican subjects during a 3.5-year follow-up [6], in 644 Chinese subjects followed up for 4.5 years [7], and in 82,069 US postmenopausal women followed up for 5.9 years [8]. In Japanese subjects, HOMA-β was significantly lower in those with type 2 diabetes than in those with normal glucose tolerance or prediabetes [9]. Moreover, in Japanese subjects, HOMA-β was lower in subjects with impaired glucose tolerance than in those with normal glucose tolerance [10]. These findings suggest that HOMA-β is a reliable indicator associated with glucose intolerance in subjects without diabetes.

Serum immunoreactive insulin (IRI) and C-peptide immunoreactivity (CPR) were measured to assess insulin secretion and were well-known markers of β cell function in the clinical setting. CPR is secreted from β-cells at an equimolar ratio to insulin, thereby reflecting endogenous insulin secretion. In contrast to IRI, CPR is not extracted by the liver [11], but excreted in the urine; therefore, CPR accurately reflects endogenous insulin secretion.

Reduction of muscle mass, sarcopenia, is a progressive disease related to frailty in elderly subjects. Because the muscle is one of the target organs of insulin, muscle mass may have an impact on glucose tolerance and should be considered when evaluating glucose metabolism. It has been reported that a reduction of muscle mass may be related to the prevalence of prediabetes [12]. However, it is not yet clear whether β-cell function is related to muscle mass, particularly in subjects without diabetes. Therefore, in the present study, we investigated the relationship between β-cell function and skeletal muscle mass in Japanese subjects without diabetes.

## Subjects and Methods

### Subjects

This study included 1098 subjects (538 men and 560 women) aged 40 to 79 years undergoing a consultation and medical examination at the Osaka Medical College Health Science Clinic between July 2013 and July 2014. Exclusion criteria were (1) diabetes (fasting glucose equal to or higher than 126 mg/dL or glycosylated hemoglobin [HbA1c] equal to or higher than 6.5%), and (2) diabetes history or treatment. This study was approved by the Ethics Committees of Osaka Medical College (No. 1315) and Osaka Medical College Health Science Clinic (No. 2011-CR-9). Written informed consent was provided by all subjects.

### Anthropometric Measurements

During the medical consultation, body height and weight were measured with the patient in the standing position. Body mass index (BMI) was calculated as body weight divided by height squared (kg/m^2^). Muscle mass was measured by bioelectrical impedance analysis (BIA) using the Body Composition Analyzer MC-190 (Tanita Corp., Tokyo, Japan) [13]. The appendicular muscle mass (AMM) was calculated as the sum of the muscle mass of the arms and the legs, and the appendicular muscle mass index (AMI) was calculated as AMM divided by height squared (kg/m^2^) [14].

### Laboratory Measurements

Blood samples were drawn on the morning of the medical consultation, after at least 10 hours’ fasting. Fasting serum glucose (FG) levels were measured by hexokinase enzymatic analysis, HbA1c level was measured by high-performance liquid chromatography, and IRI and CPR were measured by chemiluminescence enzyme immunoassay. The HOMA-β and HOMA-IR for an index of the β-cell function and insulin resistance were calculated using the following formulas: HOMA-β (%) = (IRI × 360) / (FG—63) and HOMA-IR = FG × IRI / 405, respectively.

### Statistical Analysis

IRI, CPR, HOMA-β and HOMA-IR values are expressed as medians and interquartile ranges. Other variables are expressed as means ± SD values. Differences between men and women were compared by the Student’s *t*-test. IRI, CPR, HOMA-β and HOMA-IR values were log-transformed (Log) into normally distributed values. The relationships between Log IRI and Log CPR and anthropometric and biochemical parameters (BMI, FG and HbA1c) were examined using Pearson’s correlation analysis. The relationship between clinical parameters of β-cell function (Log HOMA-β), insulin resistance (Log HOMA-IR) and age were examined using Pearson’s correlation analysis. The comparison of β-cell function (Log HOMA-β) and insulin resistance (Log HOMA-IR) among AMI tertiles was examined by using one-way ANOVA. A p-value less than 0.05 was considered significant. All analyses were performed using SPSS version 22.0 (Chicago, IL, USA).

## Results

**Fig 1:**
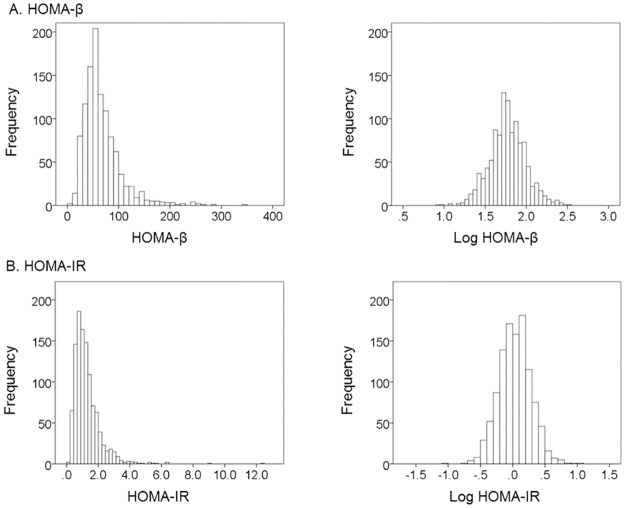
Histogram of HOMA-β and HOMA-IR with non-normal distribution and Log HOMA-β and HOMA-IR with normal distribution. HOMA-β, homeostasis model assessment of β-cell function. HOMA-IR, homeostasis model assessment of insulin resistance.

Anthropometric characteristics and laboratory measurements, including clinical parameters of β-cell function and insulin resistance, are shown in Table 1. There were no significant differences in HbA1c, Log HOMA-β, and LDL-Chol between men and women. The mean age, height, weight, BMI, AMM, AMI, FG, Log IRI, Log CPR, Log HOMA-IR, TG, AST, ALT and Cr values were significantly higher in men than in women. Conversely, mean Total-Chol and HDL-Chol values were significantly higher in women than in men. IRI, CPR, HOMA-β and HOMA-IR values were log-transformed into normally distributed values for further analysis. Fig 1 shows that the histogram of HOMA-β and HOMA-IR has a non-normal distribution while the histogram of Log HOMA-β and Log HOMA-IR has a normal distribution.

**Table 1:** Anthropometric characteristics and clinical parameters among all subjects.

	Men (n = 538)	Women (n = 560)	p-value*
Age (years)	53.0 ± 9.8	51.7 ± 8.7	0.024
Height (cm)	170.3 ± 6.3	157.5 ± 5.3	<0.001
Weight (kg)	67.8 ± 9.5	53.5 ± 8.0	<0.001
BMI (kg/m^2^)	23.4 ± 2.8	21.5 ± 3.2	<0.001
AMM (kg)	23.9 ± 3.2	16.0 ± 1.8	<0.001
AMI(kg/m^2^)	8.2 ± 0.9	6.4 ± 0.6	<0.001
FG (mg/dL)	95.8 ± 8.8	90.5 ± 7.7	<0.001
HbA1c (%)	5.5 ± 0.3	5.5 ± 0.3	0.609
IRI (μU/mL)	5.2 (3.5–7.1)	4.4 (3.2–6.2)	
Log IRI	0.7 ± 0.2	0.6 ± 0.2	0.004
CPR	1.5(1.1–1.9)	1.2 (0.9–1.5)	
Log CPR	0.16 ± 0.17	0.08 ± 0.16	<0.001
HOMA-β	57.0 (42.1–79.1)	59.2 (44.5–83.5)	
Log HOMA-β	1.8 ± 0.2	1.8 ± 0.2	0.055
HOMA-IR	1.2 (0.8–1.7)	1.0 (0.7–1.4)	
Log HOMA-IR	0.07 ± 0.25	-0.00 ± 0.24	<0.001
Total-Chol	202 ± 33	206 ± 31	0.004
HDL-Chol	60 ± 16	74 ± 15	<0.001
TG	120 ± 78	80 ± 50	<0.001
LDL-Chol	119 ± 31	115 ± 28	0.630
AST	22 ± 8	19 ± 5	<0.001
ALT	24 ± 13	15 ± 7	<0.001
Cr	0.87 ± 0.13	0.66 ± 0.14	<0.001

IRI, HOMA-β, CPR, and HOMA-IR values were expressed as median (Interquartile range). All other data are expressed as mean ± SD values.

*Student’s *t*-test.

BMI, body mass index; AMM, appendicular muscle mass; AMI, appendicular muscle mass index; FG, fasting serum glucose; HbA1c, glycosylated hemoglobin; IRI, immunoreactive insulin; HOMA-β, homeostasis model assessment of β-cell function; Log, Log-transformed; CPR, C-peptide immunoreactivity; HOMA-IR, homeostasis model assessment of insulin resistance

Table 2 shows the correlations between Log IRI and Log CPR and anthropometric and biochemical parameters (BMI, FG and HbA1c). Log IRI and Log CPR were positively correlated with BMI, FG and HbA1c in both men and women. Table 3 shows the correlation between clinical parameters of β-cell function (Log HOMA-β), insulin resistance (Log HOMA-IR) and age. In both men and women, Log HOMA-β was negatively correlated with age. Log HOMA-IR was positively correlated with age in women, however not in men.

**Table 2:** Correlation between Log IRI and Log CPR and anthropometric and biochemical parameters (BMI, FG and HbA1c) in men and women.

	Men (n = 538)		Women (n = 560)	
	r	p-value	R	p-value
BMI				
Log IRI	0.572	<0.001	0.461	<0.001
Log CPR	0.584	<0.001	0.443	<0.001
FG				
Log IRI	0.282	<0.001	0.431	<0.001
Log CPR	0.357	0.001	0.465	<0.001
HbA1c				
Log IRI	0.143	<0.001	0.132	0.002
Log CPR	0.216	<0.001	0.181	<0.001

Data are expressed as values of correlation coefficients (r). Correlation coefficients (r) and p-values were calculated using Pearson’s correlation analysis.

BMI, body mass index; FG, fasting serum glucose; HbA1c, glycosylated hemoglobin; Log, Log-transformed; IRI, immunoreactive insulin; CPR, C-peptide immunoreactivity.

**Table 3:** Correlation between clinical parameters of β-cell function (Log HOMA-β), insulin resistance (Log HOMA-IR) and age in men and women.

	Men (n = 538)		Women (n = 560)	
	r	p-value	R	p-value
Age				
Log HOMA-β	-0.152	<0.001	-0.098	0.020
Log HOMA-IR	-0.019	0.654	0.102	0.016

Data are expressed as values of correlation coefficients (r). Correlation coefficients (r) and p-values were calculated using Pearson’s correlation analysis.

Log, Log-transformed; HOMA-β, homeostasis model assessment of β-cell function; HOMA-IR, homeostasis model assessment of insulin resistance

**Fig 2:**
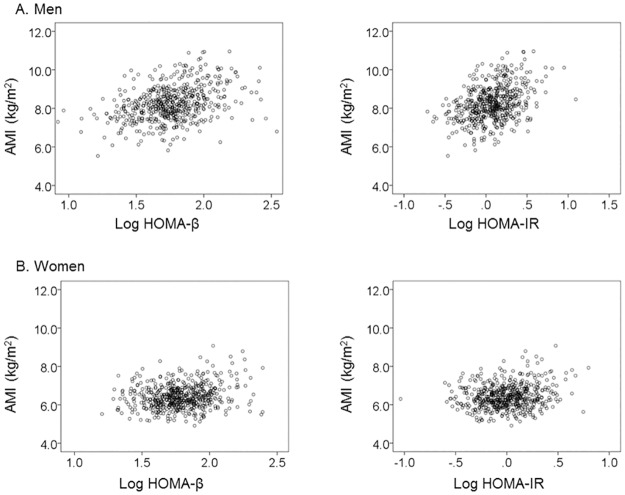
A. Correlation between AMI and Log HOMA-β (r = 0.366, p<0.001), AMI and Log HOMA-IR (r = 0.374, p<0.001) in men (n = 538). B. Correlation between AMI and Log HOMA-β (r = 0.195, p<0.001), AMI and Log HOMA-IR (r = 0.165, p<0.001) in women (n = 560). AMI, appendicular muscle mass index. HOMA-β, homeostasis model assessment of β-cell function. HOMA-IR, homeostasis model assessment of insulin resistance.

Fig 2 shows the association between AMI and clinical parameters of β-cell function (Log HOMA-β) and insulin resistance (Log HOMA-IR) in men (Fig 2A) and women (Fig 2B), respectively. In both men and women, there was a significant positive correlation between AMI and β-cell function, insulin resistance (p<0.001). We stratified subjects into tertiles, according to AMI, and evaluated associations between AMI and β-cell function (Log HOMA-β) and insulin resistance (HOMA-IR) in both men and women, respectively (Table 4). In men, Log HOMA-β and Log HOMA-IR were significantly lower in the lowest AMI tertile than in the 2nd AMI tertile and in the highest AMI tertile. Also, Log HOMA-β in the 2nd AMI tertile was significantly lower than that in the highest AMI tertile. In women, Log HOMA-β and Log HOMA-IR were significantly lower in the lowest AMI tertile than in the highest AMI tertile. Then, we divided the subjects into two groups, i.e. normal glucose tolerance (defined as fasting glucose lower than 100 mg/dL and glycosylated hemoglobin lower than 5.7%) and prediabetes (defined as fasting glucose equal to or higher than 100 mg/dL or glycosylated hemoglobin equal to or higher than 5.7%), and investigated the association between AMI and β-cell functions, insulin resistance, respectively. In subjects with normal glucose tolerance, Log HOMA-β and Log HOMA-IR were significantly lower in the lowest AMI tertile than in the middle AMI tertile and in the highest AMI tertile in men. There was no such difference in women (Table 5). In prediabetes subjects, Log HOMA-β and Log HOMA-IR were significantly lower in the lowest AMI tertile than in the highest AMI tertile in both men and women (Table 6).

**Table 4:** Associations of AMI with clinical parameters of β-cell function and insulin resistance in men and women.

	AMI		
	Lowest tertile	2nd tertile	Highest tertile
Men			
Log HOMA-β	1.67* **	1.74^†^	1.87
Log HOMA-IR	-0.03* **	0.06^†^	0.19
Women			
Log HOMA-β	1.75**	1.78	1.82
Log HOMA-IR	-0.04**	-0.01	0.03

* Statistically significant (p-value<0.05) difference between the lowest tertile of AMI and the 2nd tertile of AMI.

** Statistically significant (p-value<0.05) difference between the lowest tertile of AMI and the highest tertile of AMI.

^†^Statistically significant (p-value<0.05) difference between the 2nd of AMI and the highest tertile tertile of AMI.

AMI, appendicular muscle mass index; Log, Log-transformed; HOMA-β, homeostasis model assessment of β-cell function; HOMA-IR, homeostasis model assessment of insulin resistance

**Table 5:** Associations of AMI with clinical parameters of β-cell function and insulin resistance in men and women with normal glucose tolerance.

	AMI		
	Lowest tertile	2nd tertile	Highest tertile
Men			
Log HOMA-β	1.70**	1.75^†^	1.89
Log HOMA-IR	-0.06**	-0.02	0.14
Women			
Log HOMA-β	1.78	1.79	1.82
Log HOMA-IR	-0.06	-0.05	-0.01

* Statistically significant (p-value<0.05) difference between the lowest tertile of AMI and the 2nd tertile of AMI.

** Statistically significant (p-value<0.05) difference between the lowest tertile of AMI and the highest tertile of AMI.

^†^Statistically significant (p-value<0.05) difference between the 2nd of AMI and the highest tertile tertile of AMI.

AMI, appendicular muscle mass index; Log, Log-transformed; HOMA-β, homeostasis model assessment of β-cell function; CPR, C-peptide immunoreactivity; HOMA-IR, homeostasis model assessment of insulin resistance

**Table 6:** Associations of AMI with clinical parameters of β-cell function and insulin resistance in men and women with prediabetes.

	AMI		
	Lowest tertile	2nd tertile	Highest tertile
Men			
Log HOMA-β	1.62* **	1.73^†^	1.82
Log HOMA-IR	0.05* **	0.16	0.23
Women			
Log HOMA-β	1.70**	1.77	1.80
Log HOMA-IR	0.00**	0.09	0.14

* Statistically significant (p-value<0.05) difference between the lowest tertile of AMI and the 2nd tertile of AMI.

** Statistically significant (p-value<0.05) difference between the lowest tertile of AMI and the highest tertile of AMI.

^†^Statistically significant (p-value<0.05) difference between the 2nd of AMI and the highest tertile tertile of AMI.

AMI, appendicular muscle mass index; Log, Log-transformed; HOMA-β, homeostasis model assessment of β-cell function; CPR, C-peptide immunoreactivity; HOMA-IR, homeostasis model assessment of insulin resistance

## Discussion

In our investigation of Japanese subjects without diabetes, AMI positively correlated with HOMA-β and HOMA-IR, in both men and women. The findings of the present study suggest that appendicular muscle mass is associated with β-cell function and insulin resistance. Skeletal muscle mass decreases with age [15]. An age-related reduction of muscle mass, defined by Rosenberg in 1989, and termed sarcopenia [16], is related to physical dysfunction and quality of life [17]. In Japan, the importance of maintaining muscle mass in the aging population has been highlighted by strategies promoting physical activity [18,19] and good nutrition [20].

Some studies have reported an association between muscle mass and diabetes. Reduced appendicular muscle mass is associated with higher HbA1c levels in Japanese subjects [21]. The prevalence of pre-sarcopenia is higher in Asian Indian subjects with type 2 diabetes than in those without diabetes, independent of age [22]. These reports suggest that muscle mass may play an important role in the prevention of diabetes. Therefore, we investigated the association between muscle mass and β-cell function in subjects without diabetes.

Some studies have reported that β-cell function decreases with age. Iozzo et al. reported that β-cell function, estimated by the euglycemic insulin clamp technique, was negatively correlated with age in Europeans aged 18–85 years without diabetes (n = 957) [23]. β-cell function estimated by HOMA-β declined with age in 149 healthy Caucasians [24]. Impaired insulin secretion has been examined in 2,324 Japanese men and 1,472 Japanese women without diabetes (age 30–79 years) [25]. HOMA-β decreased with increasing age in men. Moreover, stepwise multiple regression analysis showed that age was inversely associated with HOMA-β in both sexes. However, muscle mass was not assessed. This study involved 1098 Japanese subjects without diabetes. Log HOMA-β was negatively correlated with age. These findings indicate that β-cell function decrease probably with age in subjects without diabetes.

The muscle is one of the target organs of insulin and has a significant effect on glucose tolerance. There are some reports on the association between insulin resistance and muscle mass, but few studies have examined the association between insulin secretion and muscle mass. We have reported that glucagon-stimulated CPR, following intravenous glucagon administration, was negatively associated with skeletal muscle mass in patients with type 2 diabetes younger than 65 years [26]. Over half of the patients in the study were treated with insulin and all subjects had diabetes. Tanaka et al. investigated the association between endogenous insulin secretion and muscle mass in 191 men with type 2 diabetes. They reported that decreased endogenous insulin secretion (serum CPR and IRI, and urine CPR) was an independent risk factor for sarcopenia in patients with type 2 diabetes [27]. Srikanthan et al. has found an association between increased insulin resistance and loss of muscle mass [12]. On the other hand, Lee et al. reported that AMI was positively correlated with HOMA-IR in the their report [28]. We investigated the association among AMI, β-cell function and insulin resistance in subjects without diabetes. Log HOMA-β and Log HOMA-IR were correlated positively with AMI in men and women. Analysis following stratification according to skeletal muscle mass found that in both men and women, Log HOMA-β and Log HOMA-IR were significantly lower in the lowest AMI tertile compared to the highest tertile (Table 4). Furthermore, we estimated the association among AMI, β-cell function and insulin resistance in the subjects with normal glucose tolerance and prediabetes, respectively. Log HOMA-β and Log HOMA-IR were lower in the lowest AMI tertile compared to the highest tertile. These findings indicate that decreased β-cell function is associated with reduced skeletal muscle mass in Japanese subjects without diabetes. However, in the female subjects with normal glucose tolerance, there was no association of AMI with Log HOMA-β and Log HOMA-IR (Table 5). AMI was lower in women than in men (Table 1) and the percentage of skeletal muscle mass reduction by the aging process in women was reported to be lower than that in men [29]. Therefore, the association between skeletal muscle mass and β-cell function may be unclear in women.

In our study, HOMA-β showed a non-normal distribution, as previously reported [30], however, Log HOMA-β showed a normal distribution. Thus, analysis of HOMA-β requires log-transformation of HOMA-β values, and we reported the distribution of HOMA-β in Japanese subjects as part of this study.

In the 2012 National Health and Nutrition Survey in Japan, the average height and weight for men (50–59 years) was 168.6 ± 5.7 cm and 68.0 ± 10.2 kg, respectively. For women (50–59 years), height was 156.1 ± 5.3 cm and 55.2 ± 9.2 kg, respectively. In view of the comparable average figures for height and weight between subjects in the present study and the general population in Japan, the results of the present study could be extrapolated to the Japanese population as a whole.

Our study has some limitations. Firstly, it is limited by the cross-sectional design. Further longitudinal studies are required to evaluate the association among decreased β-cell function, decreased skeletal muscle mass, and the subsequent development of diabetes. Secondly, we used HOMA-β as an index of insulin secretion, although glucose clamps and intravenous glucose tolerance tests are the gold standard for assessments of stimulated β-cell function [4]. However, in the present study, a single blood sample was taken from study subjects in the course of a consultation and medical examination, and we were not able to perform a glucose tolerance test in the subjects. Instead, we used HOMA-β to evaluate insulin secretion. It is a suitable index that can be used frequently and conveniently in clinical practice. Thirdly, we estimated skeletal muscle mass by BIA, although dual X-ray absorptiometry is the gold standard for estimating human body composition. Highly statistically significant correlations between DEXA and BIA measurements of lean body mass have been reported [31]. Thus, the BIA method is considered to be a simple, non-invasive and reasonably-reliable method, which is appropriate to perform on the subjects of the medical examination.

In conclusion, the findings of the present study indicate that decreased β-cell function evidenced by low HOMA-β is associated with reduced skeletal muscle mass in Japanese subjects without diabetes.

## References

[1] Guariguata L (2012) By the numbers: new estimates from the IDF Diabetes Atlas Update for 2012. Diabetes Res Clin Pract 98: 524–525. 10.1016/j.diabres.2012.11.006 23217268

[2] Morimoto A, Tatsumi Y, Deura K, Mizuno S, Ohno Y, et al (2013) Impact of impaired insulin secretion and insulin resistance on the incidence of type 2 diabetes mellitus in a Japanese population: the Saku study. Diabetologia 56: 1671–1679. 10.1007/s00125-013-2932-y 23680915

[3] Matthews DR, Hosker JP, Rudenski AS, Naylor BA, Treacher DF, et al (1985) Homeostasis model assessment: insulin resistance and beta-cell function from fasting plasma glucose and insulin concentrations in man. Diabetologia 28: 412–419. 3899825 10.1007/BF00280883

[4] Wallace TM, Levy JC, Matthews DR (2004) Use and abuse of HOMA modeling. Diabetes Care 27: 1487–1495. 15161807 10.2337/diacare.27.6.1487

[5] Uwaifo GI, Fallon EM, Chin J, Elberg J, Parikh SJ, et al (2002) Indices of insulin action, disposal, and secretion derived from fasting samples and clamps in normal glucose-tolerant black and white children. Diabetes Care 25: 2081–2087. 12401760 10.2337/diacare.25.11.2081

[6] Haffner SM, Kennedy E, Gonzalez C, Stern MP, Miettinen H (1996) A prospective analysis of the HOMA model. The Mexico City Diabetes Study. Diabetes Care 19: 1138–1141. 8886564 10.2337/diacare.19.10.1138

[7] Li CL, Tsai ST, Chou P (2003) Relative role of insulin resistance and beta-cell dysfunction in the progression to type 2 diabetes—The Kinmen Study. Diabetes Res Clin Pract 59: 225–232. 12590020 10.1016/s0168-8227(02)00249-8

[8] Song Y, Manson JE, Tinker L, Howard BV, Kuller LH, et al (2007) Insulin sensitivity and insulin secretion determined by homeostasis model assessment and risk of diabetes in a multiethnic cohort of women: the Women's Health Initiative Observational Study. Diabetes Care 30: 1747–1752. 17468352 10.2337/dc07-0358 PMC1952235

[9] Katsuta H, Ozawa S, Suzuki K, Takahashi K, Tanaka T, et al (2015) The association between impaired proinsulin processing and type 2 diabetes mellitus in non-obese Japanese individuals. Endocr J 62: 485–492. 10.1507/endocrj.EJ14-0611 25892189

[10] Fukushima M, Usami M, Ikeda M, Nakai Y, Taniguchi A, et al (2004) Insulin secretion and insulin sensitivity at different stages of glucose tolerance: a cross-sectional study of Japanese type 2 diabetes. Metabolism 53: 831–835. 15254872 10.1016/j.metabol.2004.02.012

[11] Polonsky K, Jaspan J, Pugh W, Cohen D, Schneider M, et al (1983) Metabolism of C-peptide in the dog. In vivo demonstration of the absence of hepatic extraction. J Clin Invest 72: 1114–1123. 6350363 10.1172/JCI111036 PMC1129279

[12] Srikanthan P, Karlamangla AS (2011) Relative muscle mass is inversely associated with insulin resistance and prediabetes. Findings from the third National Health and Nutrition Examination Survey. J Clin Endocrinol Metab 96: 2898–2903. 10.1210/jc.2011-0435 21778224

[13] Tanimoto Y, Watanabe M, Sun W, Hirota C, Sugiura Y, et al (2012) Association between muscle mass and disability in performing instrumental activities of daily living (IADL) in community-dwelling elderly in Japan. Arch Gerontol Geriatr 54: e230–233. 10.1016/j.archger.2011.06.015 21831461

[14] Janssen I, Heymsfield SB, Ross R (2002) Low relative skeletal muscle mass (sarcopenia) in older persons is associated with functional impairment and physical disability. J Am Geriatr Soc 50: 889–896. 12028177 10.1046/j.1532-5415.2002.50216.x

[15] Lexell J, Taylor CC, Sjostrom M (1988) What is the cause of the ageing atrophy? Total number, size and proportion of different fiber types studied in whole vastus lateralis muscle from 15- to 83-year-old men. J Neurol Sci 84: 275–294. 3379447 10.1016/0022-510x(88)90132-3

[16] Rosenberg IH (1989) Summery comments. Am J Clin Nutr 50: 1231–1233.

[17] Tanimoto Y, Watanabe M, Sun W, Tanimoto K, Shishikura K, et al (2013) Association of sarcopenia with functional decline in community-dwelling elderly subjects in Japan. Geriatr Gerontol Int 13: 958–963. 23452074 10.1111/ggi.12037

[18] Fujita S, Rasmussen BB, Cadenas JG, Drummond MJ, Glynn EL, et al (2007) Aerobic exercise overcomes the age-related insulin resistance of muscle protein metabolism by improving endothelial function and Akt/mammalian target of rapamycin signaling. Diabetes 56: 1615–1622. 17351147 10.2337/db06-1566 PMC2740742

[19] Frontera WR, Meredith CN, O'Reilly KP, Knuttgen HG, Evans WJ (1988) Strength conditioning in older men: skeletal muscle hypertrophy and improved function. J Appl Physiol (1985) 64: 1038–1044. 3366726 10.1152/jappl.1988.64.3.1038

[20] Katsanos CS, Kobayashi H, Sheffield-Moore M, Aarsland A, Wolfe RR (2006) A high proportion of leucine is required for optimal stimulation of the rate of muscle protein synthesis by essential amino acids in the elderly. Am J Physiol Endocrinol Metab 291: E381–387. 16507602 10.1152/ajpendo.00488.2005

[21] Sanada K, Miyachi M, Tanimoto M, Yamamoto K, Murakami H, et al (2010) A cross-sectional study of sarcopenia in Japanese men and women: reference values and association with cardiovascular risk factors. Eur J Appl Physiol 110: 57–65. 10.1007/s00421-010-1473-z 20390291

[22] Anbalagan VP, Venkataraman V, Pradeepa R, Deepa M, Anjana RM, et al (2013) The prevalence of presarcopenia in Asian Indian individuals with and without type 2 diabetes. Diabetes Technol Ther 15: 768–775. 10.1089/dia.2013.0068 23789632

[23] Iozzo P, Beck-Nielsen H, Laakso M, Smith U, Yki-Jarvinen H, et al (1999) Independent influence of age on basal insulin secretion in nondiabetic humans. European Group for the Study of Insulin Resistance. J Clin Endocrinol Metab 84: 863–868. 10084562 10.1210/jcem.84.3.5542

[24] Chiu KC, Lee NP, Cohan P, Chuang LM (2000) Beta cell function declines with age in glucose tolerant Caucasians. Clin Endocrinol (Oxf) 53: 569–575. 11106917 10.1046/j.1365-2265.2000.01132.x

[25] Oya J, Nakagami T, Yamamoto Y, Fukushima S, Takeda M, et al (2014) Effects of age on insulin resistance and secretion in subjects without diabetes. Intern Med 53: 941–947. 24785884 10.2169/internalmedicine.53.1580

[26] Shishikura K, Tanimoto K, Sakai S, Tanimoto Y, Terasaki J, et al (2014) Association between skeletal muscle mass and insulin secretion in patients with type 2 diabetes mellitus. Endocr J 61: 281–287. 24420336 10.1507/endocrj.ej13-0375

[27] Tanaka KI, Kanazawa I, Sugimoto T (2015) Reduction in Endogenous Insulin Secretion is a Risk Factor of Sarcopenia in Men with Type 2 Diabetes Mellitus. Calcif Tissue Int. 10.1007/s00223-015-9990-8 25850525

[28] Lee SW, Youm Y, Lee WJ, Choi W, Chu SH, et al (2015) Appendicular skeletal muscle mass and insulin resistance in an elderly korean population: the korean social life, health and aging project-health examination cohort. Diabetes Metab J 39: 37–45. 10.4093/dmj.2015.39.1.37 25729711 PMC4342535

[29] Metter EJ, Lynch N, Conwit R, Lindle R, Tobin J, et al (1999) Muscle quality and age: cross-sectional and longitudinal comparisons. J Gerontol A Biol Sci Med Sci 54: B207–218. 10362000 10.1093/gerona/54.5.b207

[30] Nagasaka S, Sato N, Takahashi N, Kusaka I, Ishibashi S (2007) New insights on the simultaneous assessment of insulin sensitivity and beta-cell function with the HOMA2 method: response to Caumo et al. Diabetes Care 30: e42; author reply e43. 17468370 10.2337/dc07-0016

[31] Bolanowski M, Nilsson BE (2001) Assessment of human body composition using dual-energy x-ray absorptiometry and bioelectrical impedance analysis. Med Sci Monit 7: 1029–1033. 11535954

